# Analysis of Convergent Gene Transcripts in the Obligate Intracellular Bacterium *Rickettsia prowazekii*

**DOI:** 10.1371/journal.pone.0016537

**Published:** 2011-01-26

**Authors:** Andrew Woodard, David O. Wood

**Affiliations:** Department of Microbiology and Immunology, University of South Alabama College of Medicine, Mobile, Alabama, United States of America; Louisiana State University, United States of America

## Abstract

Termination of transcription is an important component of bacterial gene expression. However, little is known concerning this process in the obligate intracellular pathogen and model for reductive evolution, *Rickettsia prowazekii*. To assess transcriptional termination in this bacterium, transcripts of convergent gene pairs, some containing predicted intrinsic terminators, were analyzed. These analyses revealed that, rather than terminating at a specific site within the intervening region between the convergent genes, most of the transcripts demonstrated either a lack of termination within this region, which generated antisense RNA, or a putative non-site-specific termination that occurred throughout the intervening sequence. Transcripts terminating at predicted intrinsic terminators, as well as at a putative Rho-dependant terminator, were also examined and found to vary based on the rickettsial host environment. These results suggest that transcriptional termination, or lack thereof, plays a role in rickettsial gene regulation.

## Introduction

The obligate intracellular bacterium, *Rickettsia prowazekii,* is the causative agent of epidemic typhus, a louse-borne disease usually associated with non-hygienic conditions arising in crowded human populations during war, famine, and as a result of extreme poverty. Due to its potential as an instrument of bioterrorism, *R. prowazekii* is classified as a category B Select Agent. The unique life style of this pathogen involves growth in the widely different environments of an arthropod louse vector and a human host. In addition, a zoonotic reservoir, the flying squirrel, has been identified in the United States [1], [2]. *R. prowazekii* pathogenicity results from the intracytoplamsic growth of the rickettsiae, leading to cell lysis and the subsequent infection of additional host cells. In *R. prowazekii*, this lifestyle is dependent on a relatively small genome that contains a high proportion of pseudogenes and non-coding sequences [3].

Rickettsial gene expression studies have focused primarily on transcription initiation. *R. prowazekii* has been shown to utilize regulated promoters and to organize genes into operons [4], [5], [6], [7], [8], [9], and rickettsial RNA polymerase was shown to exhibit properties that distinguish it from the *Escherichia coli* polymerase, such as the requirement for a supercoiled template for promoter binding, [10], [11]. However, an area of gene regulation that has not been evaluated in rickettsiae is transcriptional termination. In fact, very few functional terminators have been experimentally confirmed outside of model organisms such as *E. coli* and *Bacillus subtilis* [12]. The conservation of genes associated with the termination process (*nusA*, *nusB*, *nusG*, *mfd*, and *rho*) suggests that transcription termination has a role in rickettsial gene regulation [3]. Preliminary studies in our laboratory indicated that rickettsial termination of the *greA* gene transcript exhibited unusual properties. Transcripts of this gene were shown to extend through an intervening region and into the coding sequence of the converging *pnt* gene [13].

To gain insight into this aspect of rickettsial gene regulation, we elected to focus on areas of the rickettsial genome where gene organization suggests the most probable location for termination events; namely, the intervening regions between convergent genes [12] . Analysis of these regions revealed functional intrinsic terminators and a putative Rho-dependent terminator. Also identified were convergent genes that exhibit a lack of termination or a non-site-specific termination and the generation of rickettsial antisense RNAs resulting from the extension of transcripts into convergent gene sequences. Most importantly, we discovered that *R. prowazekii* may regulate termination efficiency of specific terminators depending on the rickettsial host.

## Materials and Methods

### Bacterial strains, host cell lines, and culture conditions

*R. prowazekii* strain Madrid E was cultured and purified from the yolk sacs of embryonated hen eggs, as described previously[14]. Purified rickettsiae were suspended in a sucrose-phosphate-glutamate-magnesium buffer solution (0.218 M sucrose, 3.76 mM KH_2_PO_4_, 7.1 mM K_2_HPO_4_, 4.9 mM potassium glutamate, and 10 mM MgCl_2_), and stored frozen at −80°C. Murine fibroblast L929 cells (American Type Culture Collection, Manassas, VA, ATCC Number CCL-1) were cultured at 34°C with 5% CO_2_ in modified Eagle's medium (Mediatech, Inc., Herndon, VA), supplemented with 10% heat-inactivated newborn calf serum (HyClone Laboratories, Logan, UT), and 2 mM glutamine (Mediatech, Inc.). For L929 cell rickettsial infections, rickettsiae propagated in hen egg yolk sacs were used to infect L929 cells at a multiplicity of infection of 5–10 rickettsiae per cell. Infected cells were planted into tissue culture flasks and incubated for 48 hours (≥90% infected and 100–400 rickettsiae per cell) before harvesting. Rickettsiae were released from the cells using ballistic shearing (mini-Beadbeater-1, BioSpec Products Inc., Bartlesville, OK). Cells were subjected to 3 cycles at the maximum agitation setting for 10 seconds with at least 10 seconds of cooling on ice between each agitation. Sheared samples were placed in a 50 ml conical vial, diluted to 30 ml with SPG-Mg, and centrifuged at 900× g for 10 minutes at 4°C. The supernatant was collected and centrifuged in a Beckman J2-21 centrifuge (Beckman Coulter, Inc., Brea, CA) at 12,000× g, 4°C for 15 minutes to pellet rickettsiae for RNA isolation.

### RNA Isolation

For RNA isolation, rickettsial pellets were solubilized using 1 ml of Tri-Reagent (Applied Biosystems, Austin, TX) per-flask equivalent for L929-grown rickettsiae or per 0.5 ml of hen egg yolk sac rickettsial preparations. Total RNA was extracted following the manufacturer's guidelines and suspended in THE RNA Storage Solution (Applied Biosystems). RNA concentration was determined using a NanoDrop ND-1000 spectrophotometer (NanoDrop Products, Wilmington, DE). Approximately 80 µg of total RNA was further purified using RNeasy-Mini columns (Qiagen Inc., Valencia, CA). Columns were treated with RNase-free DNase I (Qiagen), eluted in 100 µl (2×50 µl) nuclease-free water and treated with 1 µl of the RNase inhibitor SUPERase-In (Applied Biosystems). The quality and concentration of the RNA was determined utilizing the micro-fluidic Agilent 2100 Bioanalyzer (Agilent Technologies, Inc., Santa Clara, CA). Aliquots were immediately precipitated, re-hydrated in THE RNA Storage Solution and stored at −80°C.

### Probe Design and Production

PCR templates for probe synthesis were generated using sequence-specific primers containing T7 and T3 promoters (Integrated DNA Technologies, Inc., Coralville, IA) with *R. prowazekii* genomic DNA as template (Table S1). Amplified products were purified with Geneclean Turbo for PCR kit (MP Biomedicals, Solon, OH) and size-verified by gel electrophoresis. Radiolabled RNA probes and markers (RNA Century or Century-Plus, Applied Biosystems) were synthesized utilizing the Riboprobe T7-T3 transcription system (Promega Corp., Madison, WI). Approximately 300 ng of the purified template was added to a reaction mixture, as directed by the manufacturer, containing: Transcription Buffer (1X), 100 mM DTT, 40 U RNasin, 2.5 mM of unlabled rATP, rGTP, rCTP, 0.2 mM rUTP, 20 U T7 or T3 polymerase, and 50 µCi of [α-^32^P] rUTP (MP Biomedicals). The reaction mixture was incubated in a dry heat-block for one hour at 37°C, and immediately treated with 1 U of RQ1 DNase (Promega Corp.) for 15 minutes. Unincorporated nucleotides were removed with an RNase-free G-50 column (GE Healthcare Biosciences, Pittsburg, PA). Radiolabeled probes was then diluted to a final activity of 5.0×10^3^ cpm. Verification of a single, labeled product of the predicted size was accomplished by analyzing each labeled probe on a 5% acrylamide/8 M urea gel. Visualization was achieved using a phosphor imaging screen and detection on a Cyclone Plus phosphoimager (Perkin Elmer, Waltham, MA). Unlabeled probes were synthesized in the same manner, analyzed on a 5% acrylamide/8 M urea gel, and visualized by ethidium bromide staining. Concentrations of unlabeled probes were determined using a NanoDrop spectrophotometer.

### Ribonuclease Protection Assay

RPA analyses were conducted using the RPA III Kit (Applied Biosystems). Briefly, purified rickettsial RNA samples were pooled, separated into 50 µg aliquots, and precipitated with ethanol. Precipitated RNA was washed with 70% ethanol and dried for 4 minutes. RNA samples were then hydrated in a mixture of 3 µl of RNase-free water, 1 µl of the radiolabeled probe (5.0×10^3^ cpm/µl) and 10 µl of Hybridization Buffer III. Hybridization samples were brought to 94°C for 4 minutes and then incubated in a dry heat block overnight at 42°C. RNase digestion buffer was added to the sample, and the sample incubated for 30 minutes at 37°C. Inactivation Solution III was then added and samples were placed at −20°C for a minimum of 15 minutes. The samples were centrifuged at 16,000× g for 15 minutes, and the resulting pellet suspended in 10 µl of Gel Loading Buffer II. Experimental, and control samples as well as 5.0×10^3^ cpm of the labeled RNA Century-Plus Marker (Applied Biosystems) were incubated at 94°C for 3 minutes, analyzed by electrophoresis using a 5% acrylamide/8 M urea gel, and detected using a phosphopimager. Controls were included for each RPA analysis to confirm the validity of the assay (data not shown). Controls for each RPA analysis included: a positive control for the hybridization consisting of the labeled probe and 300 pg of its unlabeled complement. A negative control of labeled probe only, at the amount used in the experimental assay, was included to ensure that all unprotected probe would be digested. DNA contamination would be reflected by full length protection of every probe under all conditions. Therefore, the absence of fully protected probe in all assays for each RNA preparation confirmed the absence of DNA contamination. All RPA results shown are representative of a minimum of three independent RNA preparations.

## Results

### Transcriptional termination and convergent gene pairs

To analyze rickettsial transcriptional termination, we focused on the most likely location for termination, the intervening sequences between convergent genes [12]. Of the 104 convergent gene pairs annotated in the *R. prowazekii* genome [3], we selected 12 genes (Table 1), representing 6 well-separated gene pairs that, with two exceptions, met the following selection criteria. First, each gene is transcribed at detectable levels. This was evaluated using microarray data [4] or by direct measurements using RPA. In addition, in the current study intragenic positive control probes were included to confirm gene transcription (Table 1). Secondly, the gene products were detected by proteomic analysis, with the exception of RP826 and RP777. The latter gene was listed as a pseudogene in Madrid E and therefore not annotated or screened in proteomic analyses [15], [16] (unpublished results). Two of these gene pairs (RP703-RP704 and RP826-RP827) were also included due to the prediction by TransTermHP of a strong, bidirectional, intrinsic terminator within the intervening regions [12].

**Table 1:** Targeted genes and intragenic probes.

Gene	Annotation^a^	Gene Size (bp)	Probe Size^b^	Bases from Start
RP067	*parC*	2217	619	883
RP068	Unk	1323	627	628
RP145	*aspS*	1818	375	1052
RP146	Unk	1839	441	816
RP495	*glnA*	831	346	450
RP496	*rbn*	858	367	364
RP703	*ccmF*	2013	-	-
RP704	*sca5*	4932	-	-
RP777	*metK*	1143	316	105
RP778	*dnaE*	3549	399	2592
RP826	Unk	327	-	-
RP827	Unk	747	-	-

a Annotations from [3] and ERGO™ (Integrated Genomics, Chicago, IL.).

b Size in bases from NCBI.

Unk = Unknown.

Transcript detection was accomplished using ribonuclease protection assays (RPA). RPA analysis uses single-strand, labeled RNA probes that are antisense to target mRNAs. If mRNA specific to the probe is present, it will hybridize to the probe and protect it from digestion with nucleases that specifically digest single strands. The protected probe can then be analyzed by gel electrophoresis. The extent of protection allows for the estimation of termination sites and reveals a complete picture of protected transcripts within the selected region. Conversely, if the transcript does not stop and reads through the intervening region, the probe will appear as fully protected. If there are non-site specific termination events through the intervening region, multiple bands of differing sizes will be visualized. In Table 2, the sizes of the intergenic regions, the probe size, and the amount the probe overlaps the coding regions of the genes are presented for the probes used in this study.

**Table 2:** Gene pairs and intergenic probes.

Gene Pair	Annotation^a^	Intergenic Region (bp)	Probe Size	Overlap Gene A	Overlap Gene B
RP067-RP068	*parC* – Unk	341	593	122	130
RP145-RP146	*aspS* – Unk	569	791	106	116
RP495-RP496	*glnA* – *rbn*	137	512	94	281
RP703-RP704 (1)	*ccmF* – *sca5*	1910	577	370	-
RP703-RP704 (2)	*ccmF* – *sca5*	1910	833	-	-
RP703-RP704 (3)	*ccmF* – *sca5*	1910	563	-	-
RP703-RP704 (4)	*ccmF* – *sca5*	1910	665	-	293
RP777-RP778	*metK* – *dnaE*	366	682	160	156
RP826-RP827	Unk – Unk	253	466	127	86

a Annotations from [3] and ERGO™ (Integrated genomics, Chicago, IL.).

Unk = Unknown.

We assayed rickettsial RNA extracted from rickettsiae grown in hen egg yolk sacs and in L929 mouse fibroblast cells. Previous studies had indicated that mRNA assayed at 34°C from rickettsiae grown in L929 cells had a half-life of approximately 15 minutes [6], a property that would preclude the isolation of mRNA from yolk sacs due to the 4–5 hours required to isolate rickettsiae from this source. However, we found that rickettsial mRNA is present in the egg yolk sac rickettsial RNA preparation and can be detected at levels comparable to mRNA isolated from rickettsiae grown in L929 cells. The recovery of mRNA from yolk sac grown rickettsiae is most likely due to performing all manipulations at 4°C during rickettsial purification. This permitted us to assay rickettsial RNA from different rickettsial host backgrounds.

**Figure 1:**
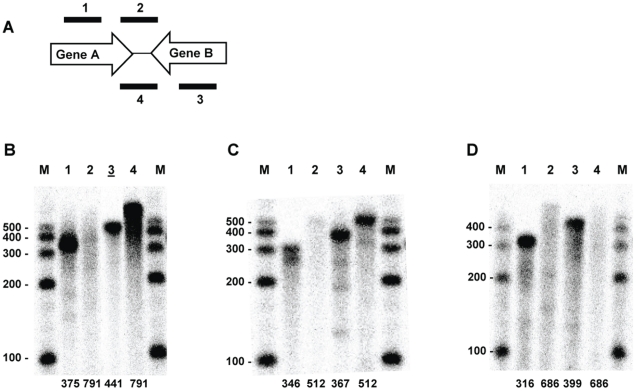
RPA analysis identifying transcripts of three convergent gene pairs that exhibit non-site-specific termination. (A) Schematic indicating the relative positions of the RPA probes for each convergent gene pair (Tables 1 and 2). The probes include two internal probes to confirm gene transcription and two intervening probes of opposite orientation to detect intergenic mRNA. Probes 1 and 2 hybridize to Gene A (left) transcripts while probes 3 and 4 hybridize to Gene B (right) transcripts. (B) RP145-RP146. (C) RP495-RP496. (D) RP777-RP778. Lane numbers correspond to the probe numbers in the schematic. Numbers at the bottom of each lane correspond to the size of the probe in bases. M, sized markers with sizes (in bases) indicated. Lane B3 (underlined) was digitally moved horizontally using Microsoft Office PowerPoint to maintain consistent lane organization.

RPA analysis of transcripts specific for three of the gene pairs is presented in Figure 1. The results reveal that the lack of specific termination sites is not an uncommon occurrence in *R. prowazekii*. For example, the RP145-RP146 convergent gene pair is transcribed as evidenced by the fully protected intragenic probes that are targeted to sites within the two genes (Fig. 1B, Lanes 1 and 3). However, RP145 transcripts do not exhibit a specific termination site within the intervening region (Fig. 1B, Lane 2). Since there is a 106 base pair overlap of the probe with RP145 (Table 2), this indicates the absence of a transcriptional stop immediately following the RP145 stop codon (a 106 base protected fragment would have been visible by gel electrophoresis). Rather, the presence of a diffuse banding pattern suggests non-site-specific termination throughout the intervening region. In contrast to the RP145 transcript, the probe targeted to the intervening region downstream of RP146 was fully protected (Fig. 1B, Lane 4) demonstrating that the RP146 transcript extends into the RP145 coding region generating antisense RNA to RP145 transcripts (the probe extends 116 bp into the coding region). The RP495-RP496 gene pair exhibited a similar termination profile: RP495 transcripts exhibit no defined termination site (Fig. 1C, Lane 2) and the probe specific for RP496 was fully protected (Fig. 1C, Lane 4). Once again this demonstrates that RP496 transcripts are extending into the RP495 coding region generating antisense RNA (the probe extends 281 bp into the coding region). The protection profile observed with the RP777-RP778 gene pair demonstrated that both gene transcripts exhibited a diffuse pattern indicative of non-site-specific termination throughout the intervening region (Fig. 1D, Lanes 2 and 4). Thus, these three convergent gene pairs of *R. prowazekii* do not terminate transcripts at specific sites within the intervening region, as might be expected based on gene orientation. In fact, read-through into the opposing gene, generating antisense RNA, is observed for two gene transcripts (Fig. 1, B, Lane 4 and C, Lane 4). These results were obtained whether the RNA was extracted from rickettsiae propagated in hen egg yolk sacs or in L929 tissue culture cells.

### Transcriptional termination at predicted terminators

To further elucidate rickettsial transcription termination, we selected two rickettsial gene pairs (RP703-RP704 and RP826-RP827) predicted by bioinformatic analysis to exhibit strong intrinsic terminators. Probes were designed to span the predicted intrinsic terminator sites of these gene pairs (Table 1). Analysis of these intrinsic terminator regions revealed a variable pattern of termination based on the source of the RNA.

**Figure 2:**
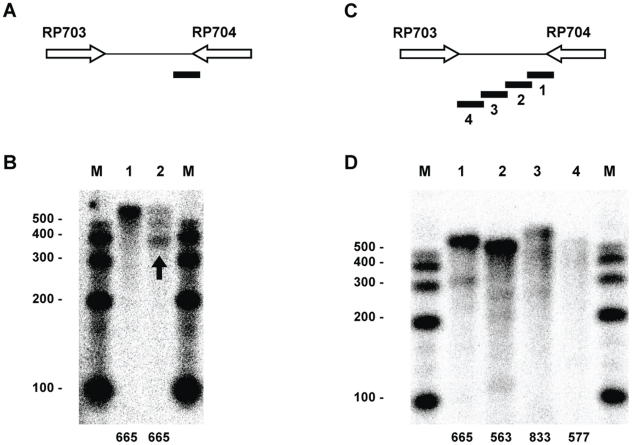
RPA analysis identifying transcripts of the RP703-RP704 intervening region. (A) Schematic indicating the relative position of the RPA probe that spans the predicted intrinsic terminator. This probe is designed to hybridize to RP704 transcripts. (B) RPA analysis with the probe indicated in A using RNA isolated from rickettsiae grown in egg yolk sacs (Lane 1) or L929 cells (Lane 2). (C) Schematic indicating the relative positions of overlapping probes spanning the intergenic region (Table 2). These probes are designed to hybridize to RP704 transcripts. (D) RPA analysis with the probes indicated in Panel B and RNA isolated from egg yolk sac grown rickettsiae. Lane numbers correspond to probe numbers in the schematic. Numbers at the bottom of lanes in B and D correspond to the size of the probe in bases. M, sized markers with sizes (in bases) indicated.

The RP703-RP704 gene pair exhibits a long intervening region of 1910 bp and a predicted strong, bi-directional terminator(5′-AAAAAAA GCCCATTTT TTC AAAGTGGGC TTTTTTT-3′) located 32 bases from the annotated end of RP704. Interestingly, the RP703 transcripts did not reach this termination site but exhibited a diffuse pattern of termination reminiscent of the transcripts presented in Figure 1 (data not shown). Thus, we were unable to evaluate the efficiency of RP703 transcript termination at the predicted site. However, functional evaluation of this site was possible for the RP704 transcript. The probe for this region was designed to extend 293 bases into RP704 and yield a protected product of 329 bases if the transcript terminated at the predicted site. A representative RPA analysis is presented in Figure 2B. When using RNA isolated from rickettsiae propagated in hen egg yolk sacs, a probe spanning the predicted intrinsic terminator was fully protected with little evidence of a stop at the intrinsic terminator (Fig. 2B, Lane 1). In contrast, the majority of the transcripts terminated at the predicted site when RNA isolated from rickettsiae grown in L929 cells was analyzed (Fig. 2B, Lane 2).

The large intervening region between RP703 and RP704 provided an excellent opportunity to examine the progression of transcription termination of transcripts that read through the predicted terminator as in rickettsiae harvested from hen egg yolk sacs. We used overlapping probes (minimum 42 base overlap) spanning the entire intervening region of 1910 bp and extending 370 bases into the RP703 gene. Probes 1 and 2 were essentially fully protected (Fig. 2D, Lanes 1 and 2) indicating complete read-through. Less full-length probe was detected for probe 3 (Fig. 2D, Lane 3), and only a negligible amount of full-length probe 4 (Fig. 2D, Lane 4) was detected. This is another example of the absence of a distinct termination site and an incremental, non-site-specific termination.

**Figure 3:**
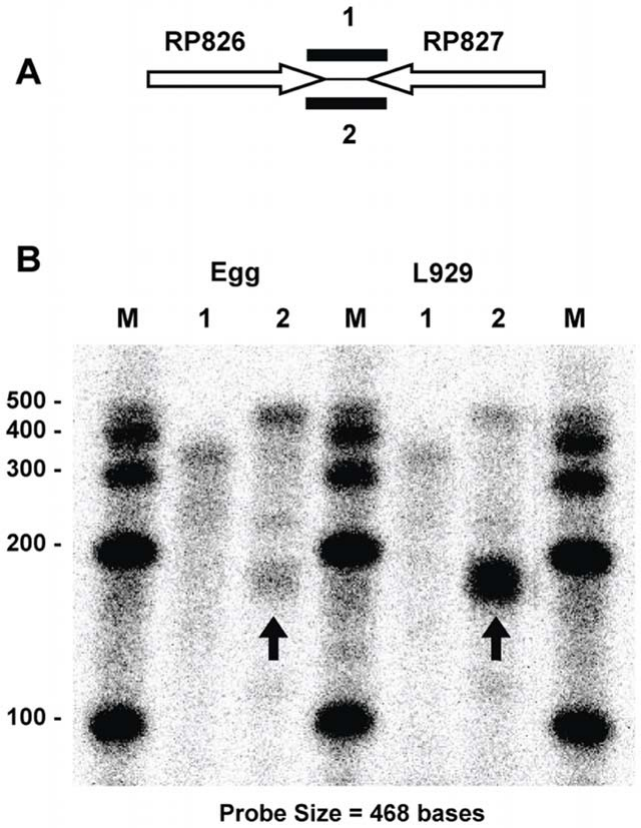
RPA analysis identifying transcripts of the RP826-RP827 intervening region. (A) Schematic indicating the relative position of the RPA probes that span the intergenic region. Probe 1 is designed to hybridize to RP826 transcripts while probe 2 hybridizes to RP827 transcripts. (B) RPA analysis of rickettsial RNA isolated from rickettsiae grown in egg yolk sacs or in L929 cells. Lane numbers correspond to probe numbers in the schematic. M, sized markers with sizes (in bases) indicated. The marker lane on the right was digitally moved horizontally using Microsoft Office PowerPoint to maintain consistent lane organization. Arrows identify the band resulting from termination of RP837 transcripts at the predicted terminator.

Differential termination at an intrinsic terminator, based on RNA source, was not unique to the RP703-704 gene pair. RP826-RP827 was an additional convergent gene pair with a predicted strong, bidirectional, intrinsic terminator (5′-AAAAA GGGTCTTTA TTAA TAAAGACCC TTTTT) that exhibited a similar termination profile (Fig. 3). Interestingly RPA analysis of RP826 transcripts revealed no difference between RNA obtained from hen egg yolk sac grown rickettsiae and L929 cell grown rickettsiae (Fig. 3B, Lanes 1). The majority of the transcripts correspond to the predicted size for termination at the intrinsic terminator (347 bases). In contrast, there was a dramatic difference in the ratio of terminated versus read-through transcripts for RP827 depending on the rickettsial host (Fig. 3B, Lanes 2). Once again the majority of transcripts detected in RNA from hen egg yolk sac grown rickettsiae completely protected the full-length probe indicating transcription through the termination site and extension of the RP827 transcript into the coding sequence of RP826. The opposite result was observed for RNA isolated from rickettsiae grown in L929 cells where the majority of the RP827 transcripts protected a 187 base portion of the probe indicating termination at the predicted terminator site. Thus, *R. prowazekii* exhibits intrinsic terminators that appear to function efficiently in rickettsiae grown in L929 tissue culture cells but are predominantly bypassed in rickettsiae growing in hen egg yolk sacs.

### Identification of a putative Rho-dependent termination site

**Figure 4:**
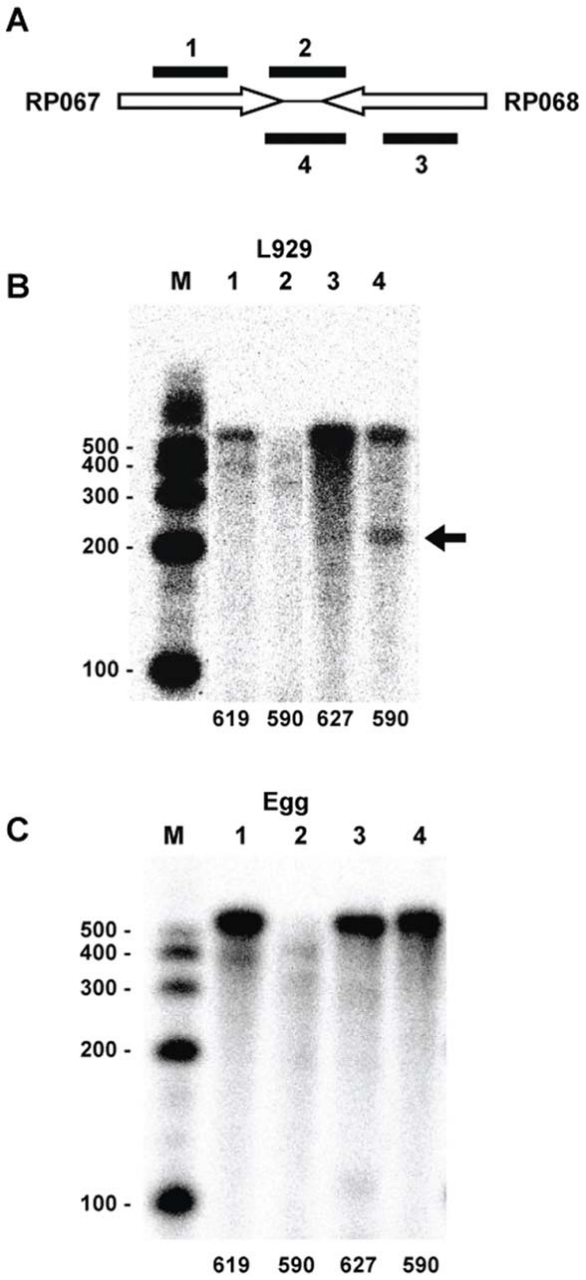
RPA analysis characterizing transcripts of the RP067-RP068 gene pair region. (A) Schematic indicating the relative positions of the RPA probes targeted to the convergent gene pair. The probes include two internal probes to confirm gene transcription and two intervening probes of opposite orientation to detect intergenic mRNA. Probes 1 and 2 hybridize to RP067 transcripts while probes 3 and 4 hybridize to RP068 transcripts. (B) RPA analysis of RNA isolated from rickettsiae harvested from L929 cells. Lanes were digitally moved horizontally using Microsoft Office PowerPoint to maintain consistent lane organization. (C) RPA analysis of RNA isolated from rickettsiae harvested from egg yolk sacs. Lane numbers correspond to probe numbers in the schematic. Numbers at the bottom of each lane in B and C correspond to the size of the probe in bases. M, sized markers with sizes (in bases) indicated. The arrow indicates a band resulting from specific termination of RP068 transcripts.

The intervening region between RP067 and RP068 does not contain a predicted intrinsic terminator. However, we detected a specific termination site by RPA for the RP068 message when using RNA isolated from rickettsiae grown in L929 cells (Fig. 4B, Lane 4). Similar to the results of gene pairs described in Figure 3 above, RP067 transcripts display a banding pattern indicating non-site-specific termination extending through the intervening region (Fig. 4B, Lane 2). Once again, when we analyzed transcripts from these genes using RNA isolated from rickettsiae grown in hen egg yolk sacs, a very different pattern was observed for the RP068 transcripts (Fig. 4C, Lane 4). No evidence of termination was observed. The entire probe was protected indicating read-through of the transcripts into the RP067 coding region. The lack of a predicted intrinsic terminator and the differentially regulated partial termination at this site suggests that the detected terminator is a Rho-dependent terminator. While we suspect that this is a Rho-dependent terminator, sequences associated with such terminators are not as easily identifiable as those of intrinsic terminators [17], and we were unable to confirm this identification.

A summary of the transcriptional results for all the genes examined in this study is presented in Table 3.

**Table 3:** Summary of Gene Termination Events.

Gene	Annotation^a^	Egg	L929
RP067	*parC*	NST^b^	NST
RP068	Unk	R	R/ST
RP145	*aspS*	NST	NST
RP146	Unk	R	R
RP495	*glnA*	NST	NST
RP496	*rbn*	R	R
RP703	*ccmF*	NST	NST
RP704	*sca5*	R	ST
RP777	*metK*	NST	NST
RP778	*dnaE*	NST	NST
RP826	Unk	ST	ST
RP827	Unk	R	ST

a Annotations from (1) and ERGO™ (Integrated Genomics, Chicago, IL.).

b R = Read through into opposing gene, ST = Specific Termination,

NST = Non-Specific Termination.

Unk = Unknown.

## Discussion

Until now termination of transcription in *R. prowazekii* has received little attention. Analysis of the *R. prowazekii* genome reveals a highly reductive genome containing many pseudogenes and a high proportion of non-coding regions. However, the retention of genes involved in the termination process (*nusA*, *nusB*, *nusG*, *mfd*, and *rho*) suggests that transcription termination has a role in rickettsial gene regulation. By targeting multiple areas hypothesized to be regions of termination, this study provides the first comprehensive analysis of transcription termination in this pathogen.

Examination of the *R. prowazekii* genome identified the presence of stem loop structures preceding oligo (T) sequences typical of bacterial termination sites. As demonstrated by our results with two predicted terminators found in the intervening regions of the RP703-RP704 and RP826-RP827 gene pairs, such classical terminators do function in *R. prowazekii*. However, one characteristic of rickettsial transcriptional termination evident from these studies is the absence of site-specific termination within many intervening regions. Of the 12 gene transcripts analyzed, all but one (RP826) exhibited either a putative non-site-specific termination or complete read-through of the intervening region that extended into the opposing gene coding sequence. This suggests that specific termination may not be prevalent in this relatively slow-growing, obligate. intracellular bacterium. The presence of protein products from the converging genes demonstrates that the absence of specific termination and the subsequent extension of transcript into the convergent gene do not impede nascent mRNA synthesis to a point affecting the survival of the rickettsiae. The exceptions may be those intrinsic terminators whose efficiencies appear to be regulated.

The efficiency of an intrinsic terminator in *E. coli* can be modulated as various factors (e.g. NusA) interact and influence the transcription complex [17]. We have identified similar alterations of terminator efficiency in *R. prowazekii*. RNAs isolated from rickettsiae grown in different host cell environments exhibited regulated transcription termination. Interestingly, rickettsial growth in the hen egg yolk sac generated RNAs that, in all but one case (RP826), failed to terminate at an intrinsic termination site, possibly reflecting a more suitable rickettsial growth environment. Although increased transcription might be assumed to require more specific termination to prevent a negative impact on convergent genes, the need to prevent wasteful transcription may assume priority if rickettsiae are not in an optimum environment. Alternatively, the need to initiate termination for appropriate gene expression during stress has been demonstrated during oxidative stress in *Caulobacter crescentus* [18]. Proteomic studies in our laboratory detect more stress-associated proteins in rickettsiae propagated in tissue culture cells than in rickettsiae grown in hen egg yolk sacs (unpublished data).

An alternative explanation for the non-specific termination of transcripts detected between some of the convergent gene pairs is the presence of RNase activity that could fragment a single transcript into many smaller fragments. Our RPA analyses cannot distinguish between transcripts generated by non-specific termination or by RNase digestion. However the presence of specific transcripts generated at predicted termination sites, suggests that RNAse activity on these transcripts is minimal. This coupled with the presence of long transcripts (e.g. RP704) extending far into the intervening region, once again suggests that RNase digestion is not the sole explanation for the observed transcripts.

The presence of rickettsial antisense RNAs generated by the lack of termination between convergent genes was an intriguing finding. The existence of antisense RNA was evident from the complete protection of probes that extended, in some cases, hundreds of bases into convergent genes. While the presence of antisense RNA in bacteria is not uncommon, most such RNAs are associated with specific small regulatory RNAs or with intergenic promotion rather than termination [19], [20]. Recently, examination of the *Helicobacter pylori* transcriptome revealed the widespread occurrence of antisense transcripts leading to the speculation that some of these may be due to imperfect termination [21]. These results are similar to an earlier study in *E. coli* that detected extensive antisense transcription throughout the genome [22]. Using a whole genome tiling microarray analysis, antisense transcripts have also been identified in the obligate intracellular bacterium, *Anaplasma phagocytophilium* [23]. Interestingly, in contrast to the *E. coli* and *H. pylori* results and our identification of several examples of antisense transcripts generated by read-through into convergent genes, the *A. phagocytophilum* whole genome study identified only one gene, p44, associated with this phenomenon [23]. Bacterial antisense RNAs have been shown to regulate gene expression [20]. The common occurrence of transcriptional read-through into convergent genes and the regulation of terminator function suggests a role in rickettsial intracellular survival and growth.

## Supporting Information

Table S1 Primer sequences used to generate RPA probes.
(DOC)
