# Supplementary material for: Analysis of Convergent Gene Transcripts in the Obligate Intracellular Bacterium Rickettsia prowazekii
Source: PLoS One. 2011 Jan 26;6(1):e16537. doi: 10.1371/journal.pone.0016537 (PMC3027695; doi:10.1371/journal.pone.0016537)
Supplement: Table S1 — Primer sequences used to generate RPA probes. (DOC) [file pone.0016537.s001.doc]

| **Table S1. Primer sequences used to generate RPA probes** | | | |
| --- | --- | --- | --- |
| **Identifier^a^** | **Gene** | **Primer^b^** | **Gene Specific Sequence (5’-3’)** |
| NP_220461.1 | RP067 | T3 | acttgtacgccttgctcc |
|  |  | T7 | ccgatattataagggtgg |
| NP_220462.1 | RP068 | T3 | ctcctgatacagctaatactgg |
|  |  | T7 | gcatatggtaataacccaggc |
|  | RP067-068 | T3 | gcctgggttattaccatatgc |
|  |  | T7 | gttggaatgatggcac |
| NP_220536.1 | RP145 | T3 | caattccaccttgtggc |
|  |  | T7 | ggggcaagaggtcttggc |
| NP_220537.1 | RP146 | T3 | cggaatcttgtttgggtc |
|  |  | T7 | gataatgctgtaacgctagc |
|  | RP145-146 | T3 | caaagcacacaacaac |
|  |  | T7 | gcttatgaatgccccaagc |
| NP_220871.1 | RP495 | T3 | ggatcgaacgcgttcc |
|  |  | T7 | gcagcacaagggatatg |
| NP_220872.1 | RP496 | T3 | ctccaccaccttacataag |
|  |  | T7 | ccaagagaaccatacatgag |
|  | RP495-496 | T3 | cgttgtattatatattgcc |
|  |  | T7 | cgggaacgcgttcgatcc |
| NP_221063.1  NP_221064.1 | RP703-704 | T3 | cgtattacttcataaaatgagg |
|  |  | T7 | ggatttcattggagaagttggc |
|  | RP703-704 | T3 | gctttgatctctatattgcgc |
|  |  | T7 | cctcattttatgaagtaatacg |
|  | RP703-704 | T3 | ggcaaattggatatacattacg |
|  |  | T7 | gcgcaatatagagatcaaagc |
|  | RP703-704 | T3 | gctaaagtagctggtagtactg |
|  |  | T7 | cgtaatgtatatccaatttgcc |
|  | RP777 | T3 | gcctagtaaataggtgcaggc |
|  |  | T7 | cctaatgggcgtgttgcttg |
| NP_221128.1 | RP778 | T3 | gcaaaagatactgcggttaagcc |
|  |  | T7 | ccctgatattcagtaagaggatga |
|  | RP777-778 | T3 | gcatcgcgcactagtaatgg |
|  |  | T7 | gctcataacagagcatttcg |
| NP_221175.1  NP_221176.1 | RP826-827 | T3 | aaaaccattcatggggttca |
|  |  | T7 | tgctactgcagcagaattacct |

^a^ NCBI gene identifiers for rickettsial genes; RP777, originally annotated as a pseudogene, and the intergenic regions lack specific identifiers

^b^  T3 primers are preceded by the T3 sequence 5’-aattaaccctcactaaaggg-3’ while the

T7 primers are preceded by the T7 sequence 5’-taatacgactcactatagggc-3’
